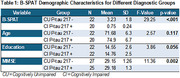# Brief Cognitive Challenge Tests for Screening at Risk Older Adults

**DOI:** 10.1002/alz70857_101161

**Published:** 2025-12-25

**Authors:** Elizabeth A. Crocco

**Affiliations:** ^1^ University of Miami Miller School of Medicine, Miami, FL, USA

## Abstract

**Background:**

Emerging plasma biomarkers for Alzheimer's disease (AD) are increasingly used in primary care. However, their clinical utility remains limited without accompanying cognitive assessments. This study introduces the Brief Semantic Paired Associates Test (B‐SPAT), a novel, three‐minute computerized cognitive test that holds potential for primary care settings.

**Method:**

We evaluated 22 cognitively unimpaired (CU) participants who were plasma *p*‐Tau217‐ and 20 participants with mild cognitive impairment (CI) who were plasma *p*‐Tau217+. The B‐SPAT is a fully digitized, six‐item semantic paired‐associate learning task, where participants match a stem word (e.g., a bird) to a non‐bird target, creating a cognitive challenge through competing associations.

**Result:**

After adjusting for MMSE and Hispanic/Latino ethnicity, B‐SPAT remained statistically significant [F(1,38) = 15.64; p < .001]. Logistic regression analysis showed that B‐SPAT's sensitivity was 90.0%, specificity 86.4%, and overall correct classification 88.1%. This outperformed the MMSE (sensitivity = 65.0%, specificity = 72.7%) and the delayed paragraph recall from the National Alzheimer's Coordinating Center (NACC) Uniform Dataset version 3 (sensitivity = 70.0%, specificity = 77.3%).

**Conclusion:**

The B‐SPAT is a promising, brief digital tool that can complement plasma AD biomarkers for screening in primary care and community settings.